# Assessing the relationship among service quality, student satisfaction and loyalty: the NIGERIAN higher education experience

**DOI:** 10.1016/j.heliyon.2021.e07590

**Published:** 2021-07-15

**Authors:** Taiye T. Borishade, Olaleke O. Ogunnaike, Odunayo Salau, Bolanle D. Motilewa, Joy I. Dirisu

**Affiliations:** Department of Business Management, Covenant University, PMB 1023, Ota, Ogun State, Nigeria

**Keywords:** Service quality, Satisfaction, Loyalty, Student, Higher education

## Abstract

Higher educational institutions are engaged in the provision of services and thus require better focus on satisfying the needs and anticipation of their participating consumers (students). Additionally, it is the delivery of quality services that creates loyal consumers: consumers who patronize the institution more and who stimulate others to patronize. While past researchers have discovered a relationship between service quality and student loyalty in higher educational institutions (HEIs) in developed countries, the peculiar nature of HEIs operating in an emerging country like Nigeria is yet to be examined. Therefore, this study examined the role of quality service, student satisfaction and loyalty in higher education institutions in Nigeria. The study was conducted in a private University in Nigeria because of the stringent competition within the subsector. The descriptive and inferential statistics were employed in this study. A survey of 265 students from the private university provided data for the study through structured questionnaire. Utilizing the structural equation model. The findings revealed a significant association between service quality and student loyalty. However, this relationship is mediated by student satisfaction. Going by the discoveries of the study, it is suggested among others, that the delivery of quality services should be targeted towards satisfying the student as this will help build the loyalty of the student to the institution.

## 1 Introduction

The number of higher educational institutions (HEI) in the world today has increased exponentially, thus leading to higher competition (Hemsley-Brown and Oplatka, 2006). To compete effectively, institutions are consequently adopting marketing concepts that portray students as customers, and ensuring all strategies are targeted at increasing student enrolment. Students search for institutions that are capable of delivering exceptional, unforgettable and individual educational experiences (Conefrey, 2018). Furthermore, as consumers, students search for educational platforms that will develop the capacity needed for lucrative careers. Thus, HEIs are re-engineering their operations in such a way that they focus more on competitive educational activities that are centred on quality evaluation (De Jager and Gbadamosi, 2013). However, in the developing country's perspective, it is argued that higher education service quality still falls below accomplishing the global standard level, especially in emerging nations such as Nigeria (Olokundun et al., 2019; Hemsley-Brown and Oplatka, 2006). It is argued that given the rise in number of higher institutions in the country, HEIs must take note of the student rising expectations and focus on advancing the quality of educational service provision in order to remain competitive. It is further stipulated that delivering a higher degree of quality reduces costs and maintains satisfied consumers, and eventually produces greater profit margins for any institution (Abdullah, 2005, 2006; Cronin and Taylor, 1994; Parasuraman et al. 1985, 1988, 1991; Sultan and Wong, 2010; Voon, 2009). In Nigeria, for several years, the higher education sector has suffered severe neglect from government and financial institutions, thus leading to a steep decline in the value of services provided by HEIs. However, with the emergence of private HEIs in the country, there appears to be an improvement in service quality and contentment, which is evident in the top quality of graduates from these private universities, thus leading to higher competition within HEIs in Nigeria. Given the above, it becomes pertinent to ascertain a relationship between service quality, student satisfaction and loyalty in HEIs in Nigeria. In creating a link between these variables, the “SEVQUAL” theory by Parasuraman et al. (1988) is adopted. Therefore, this paper espoused the Parasuraman et al. (1988) and Zeithaml et al. (2009) “five components of service quality, which are tangibility, responsiveness, reliability, assurance and empathy”. These dimensions are expended to advance the hypotheses of this study. Thus, the objectives of the paper are to:

I. Determine the direct effect of the measurements of service quality on students' loyalty in the tertiary education institution of Nigeria
II. Investigate the mediating effect of students' satisfaction in the relationship amid service quality and students' loyalty

### 1.1 Literature review

#### 1.1.1 The concept of service quality

According to Zeithaml, and Bitner (2000), a service can be defined as every business endeavour rendered by an individual party to another party, that is essentially intangible and via exchange that satisfies a recognized need and want. As a concept, quality is conceived from the standpoint of products/services in business, and it has been observed that it is most challenging to develop the concept of quality standard in service sector because of the intangibility nature or features of service (Küçükaltan, 2007). According to Yilmaz (2007), service quality can be concisely referred to as an experience related to customers' anticipations and perceptions of the service delivered. Therefore, if the delivered service does not match or surpass customer anticipations, the quality of service will be perceived as low, but if it surpasses customer's anticipations, the quality of service will be perceived as high (Akbaba and Kilinc, 2001).

Parasuraman et al. (1988) developed various components organisations can employ to appraise service quality, which is mostly cited by researchers in the appraisal of service quality. They used the 10 dimensions comprising physical/tangible features, courteousness responsiveness, security/safety, competence, credibility, reliability, communication, convenience and understanding with the customer and subsequently advanced the SERVQUAL measure which comprises 22 dimensions in five measurements. These measurements according to Parasuraman et al. (1988) and Zeithaml et al. (2009) include:

i. Tangible/Physical characteristics: The exterior or look of structures, equipment and tools, and the employees in the course of service delivery.
ii. Reliability: The capacity of the organisation to deliver services in a suitable and reliable way as assured.
iii. Responsiveness: Readiness to aid the consumer and to deliver provide quick service,
iv. Assurance: The capacity of the service provider to be well mannered, well informed and capacity to produce a feeling of self-confidence in the consumers.
v. Empathy: The ability of the organisation to see itself as the customer, gives personal attention to consumers, and displays a particular interest in consumers. Therefore, HEI must embrace and manage service quality to be relevant in the competitive environment.

#### 1.1.2 Service quality, students’ satisfaction and loyalty in HEI

Researchers have classified service quality as the foremost driver of student gratification and the eventual result is customer loyalty (Cronin et al., 2000; Petterson and Spreng, 1997). Currently, the notion of quality service in tertiary education institution is regarded very significant for the student, when deciding on the university they wish to patronize. According to Tan and Kek (2004) the quality of education is ascertained by the degree to which the students' desires and expectations are fulfilled. Quality education can be seen as a series of descriptions in a study package and how it is being provided to meet the expectation of the recipients (Korka, 2009). Students who regarded quality education very high are likely going to show a positive behavioural intention towards the institution (Frances, 1995). Nowadays, students are more judgemental concerning the delivery of quality education compared to how it was in the past (Worlu et al., 2016). Thus, student's satisfaction evaluation is considered vital for higher education managers when establishing strategic objectives (Oldfield and Baron, 2000). Different scholars have contended that service quality is a forerunner of students' gratification (Ogunnaike et al., 2018; Zeithaml et al., 2009; Parasuraman et al., 1988) According to MarzoNavarro et al. (2005) student satisfaction is a multifaceted notion, involving various dimensions. Elliott and Shin (2002) also assert that student gratification is the positivity of student's personal appraisal of the several consequences and involvements connected with their schooling. Student satisfaction shows the extent to which student expectations are achieved. The fierceness of competition in higher education has forced higher educational institutions to deliver exceptional learning experiences to obtain a greater market share in the sector (Curtis et al., 2009). Based on the level of this competition, modern day tertiary institutions pay more attention to learner satisfaction (De Jager and Gbadamosi, 2013). Quintal and Phau (2016) discovered that student loyalty is an indication of learner gratification and service quality in education is indirectly connected to learner loyalty.

A study was piloted in German Universities with the intention of finding the connection between quality and student loyalty and it was discovered that teaching quality and students' responsive obligation to their universities were vital for their loyalty (Hennig-Thurau et al., 2001). A study was also carried out on Spanish University undergraduates; the outcomes showed that university image inspires the undergraduate satisfaction with the institution (Palacio et al., 2002). DeShields et al. (2005) combined the Herzberg's two factor notion with a satisfaction model to scrutinise the elements that impact the satisfaction of student with education and they discovered that, the competence of the academic staff and the quality of lecture delivery were the main factors that influenced the quality of their experience and satisfaction. Ajzen and Fishbein (1980) postulated that a consumer's intentions and actions could be predicted by attitudes. Based on the theory, the student satisfaction and loyalty assumes that student attitude influences the intentions to remain in the university (Ibidunni et al. (2021). Service quality as a concept is equally related with student loyalty in higher education institution (Ogunnaike et al., 2014). Mustaffa et al. (2016) discovered that the quality of service leads to improved market coverage and recurrent transactions that eventually result in customer loyalty. However, some scholars are of the opinion that students' satisfaction instead of service quality exercises greater effects on purchase intentions of the students (Elliott and Shin, 2002; Cronin and Taylor, 1992). While other scholars equally delivered robust empirical confirmations reinforcing that, the quality of service promotes students' intentions to stay with their institution.

#### 1.1.3 Study hypotheses

I. There is no significant effect amid the measurements of service quality namely: (a) responsiveness (b) reliability (c) tangibility (d) empathy and (e) assurance and students' loyalty.
II. There is no substantial mediating effect of students' satisfaction in the relationship between quality of service and students' loyalty

## 2 Theoretical model

**Figure 1:**
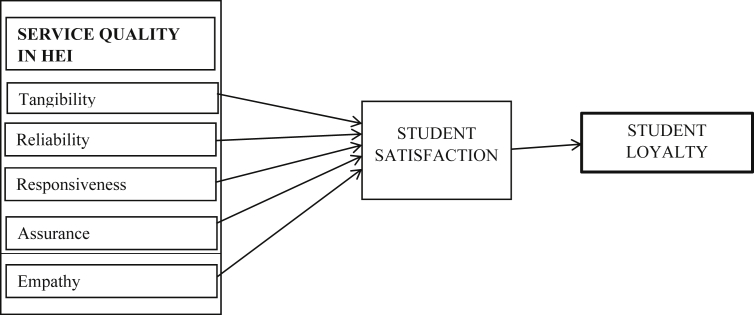
Conceptual model. Source: Conceptual model of the study.

The authors offer a conceptual model developed based on a review of literature indicating the link between service quality, customer satisfaction and student loyalty as shown in Figure 1:

Higher Education Institutions can satisfy their students and make them loyal to the institution via proper service quality application of responsiveness, reliability, tangibility, empathy and assurance, therefore, when HEI deliberately and meritoriously makes service quality a strategic focus, it principally generates a lifelong consciousness in the memory of the students and make them satisfied. This satisfaction generate into loyalty to the HEI. Therefore, conscious service quality is imperative for HEI to sustain and create student loyalty.

### 2.1 Methods

The primary intention of this paper is to assess the connection amid quality service, student satisfaction and students loyalty in an advanced education institution of Nigeria. The service quality was determined utilising the SERVQUAL measurement projected by Parasuraman et al. (1988) and Zeithaml et al. (2009), which are responsiveness, assurance reliability, tangibility and empathy. A descriptive study design was employed for this paper and the reason for the descriptive design is based on the point that it concentrated on the occurrence of interest, which is meant to give concise and scientific responses to the questions on the different evaluations of variables.

### 2.2 Sample size and sampling procedure

This study population comprised the final year student of a private university ranked number one in Nigeria by the Times Higher Education (THE, 2019). The sample size of 320 respondents were established from a population of 1610 final year students of the university based on Yamane's formula (Yamane, 1967)

n = N/(1+ N (e)^2^)

Where: n- sample size.

N- Population of student

e = Accepted error limit 0.05 (5%) on the basis of 0.95% (95%) confidence level.

Calculation:

n = 1610/1 +(1610)0.05^2^

n = 1610/1 + (1610)0.0025

n = 1610/5.025

n = 320.39 respondents

approximately = 320 respondents.

320 copies of self-administered questionnaire were distributed, 265 copies of questionnaire were returned, which amounts to a response rate of 82.5%. The inquiry form was dispensed to the undergraduate student immediately after a compulsory general lecture for all final year students. These copies of the survey instrument were personally administered by the investigator and two qualified research aides and were retrieved immediately. This indicates that the samples are assorted as it involves representatives from different departments of the university. Structural Equation Modelling (SEM) using IBM SPSS Amos 24 was used to examine the relationship between service quality and student loyalty. The substantial values of the test showed that there is no statistically substantial variance amid the groups’ involved (significant p > 0.05) (Ary et al., 2006).

**Inclusion/Exclusion criteria:**

Participants must be final year students while the Lecturers in the class were excluded.

### 2.3 Validity and reliability of the research instrument

**Figure 2:**
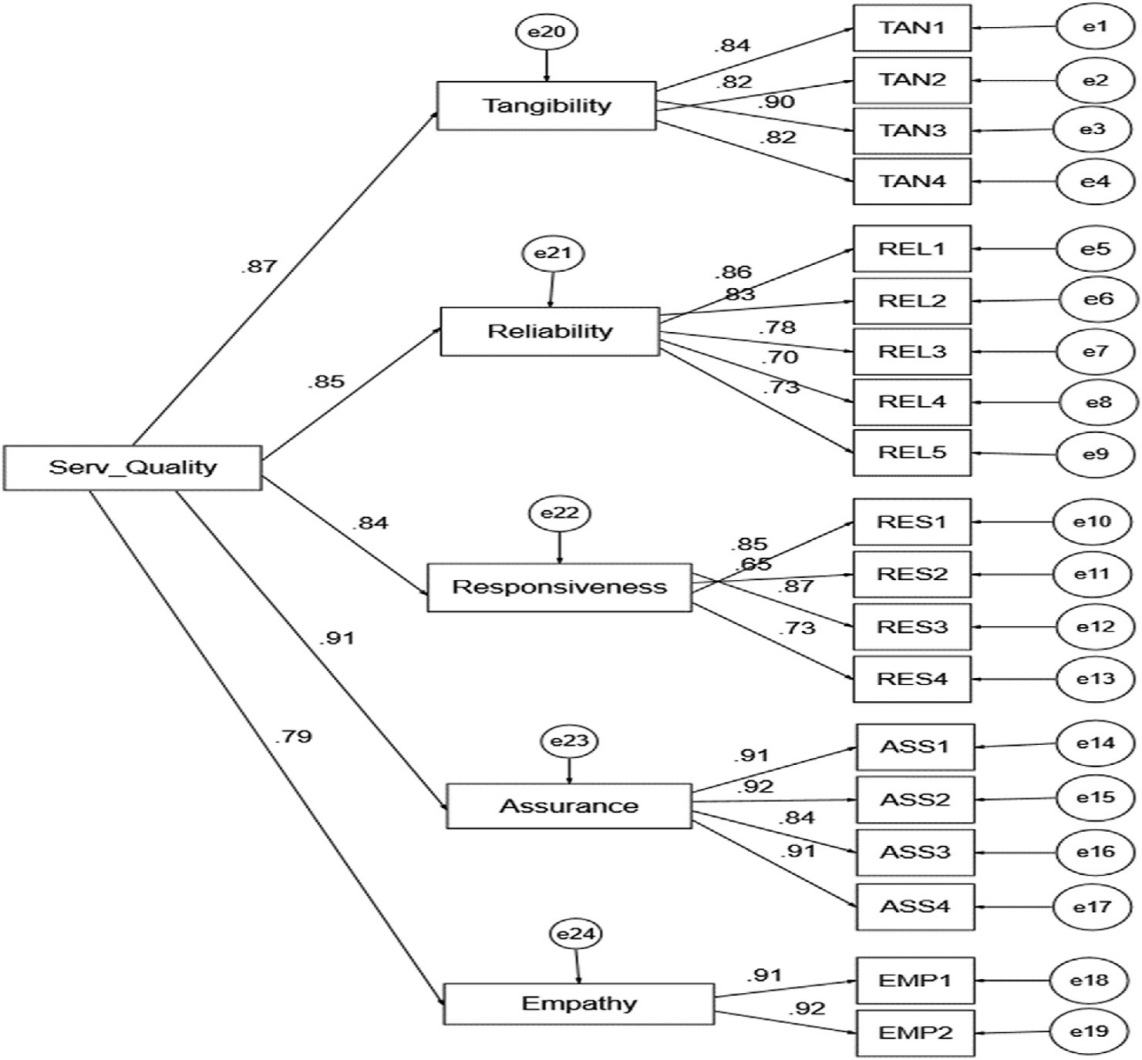
Confirmatory Factor Analysis (CFA) loadings.

This study embraced the method proposed by Anderson and Gerbing (1998) to appraise model of fits, construct validity and the hypotheses of the paper. The method espoused: (1) measurement pattern and (2) Structural Equation Model (SEM). Both models in the paper have hypotheses and dimension objects that fulfil construct validity (convergent validity). To validate the initial step of measurement pattern, the study employed Confirmatory Factor Analysis (CFA) as presented in Figure 2 to estimate the consistency of the item, item loadings, combined consistency, convergent validity, and miscalculation variance. The three situations are, firstly, the CFA loadings indicated that all measurement and scale objects are important and surpassed the smallest value standard of 0.70. Secondly, every hypothesis combined reliability surpassed 0.80. Thirdly, each hypothesis's average variance extracted estimated (AVE) exceeded 0.50 as obtainable in Figure 2 and Table 1 respectively. Meanwhile, the following measures of Goodness-of-Fit Index (GFI) as recommended by Fornell and Larcker (1981), which are chi-square/degree of freedom (χ^2^/df), Tucker Lewis Index (TLI), Standardized Root Mean Residual (SRMR) Comparative Fit Index (CFI), and Root Mean Square Error of Approximation (RMSEA) (Table 3) were also considered in the analysis.

### 2.4 Ethical consideration

The principal investigator had submitted to the Business Management Research Ethics Committee for ethical approval of the study proposal (Approved No: BMREC 05/238). A letter of introduction was given to the research team which was presented to the coordinator of the general course lecturer stating the purpose of the research. It is also worth noting that the course coordinator gave his or her verbal agreement for this study. The application letter also included a research ethics approval form. This type of research is categorized as exempt research that involves a survey with no or minimal risk i.e. level 1 research as presented in the Research Ethical Application Form. In the spirit of anonymity and confidentiality, exempt research work in management sciences does not require signed consent from the participants but implied consent is usually enough. The researchers ensured that the respondents were properly informed on the background and objective of the study, as well as kept up to date on the participation process, by obtaining verbal consent. The study's participants (students) were all properly informed about their right to reject or participate, which provided them more confidence in expressing their agreement. Respondents' right to privacy and free will were both identified as ethical issues.

## 3 Results and discussion

The outcomes of CFA investigation indicate that the factor loadings for every main variable array between 0.646 and 0.924. The merging validity conveyed for the in constants are as follows: tangibility = 0.909, responsiveness = 0.861, reliability = 0.887, assurance = 0.941, and empathy = 0.914. The three (3) situations employed to evaluate merging validity as supported and proposed by Bagozzi and Yi (1988) and Fornell and Larcker (1981) are met. Firstly, the CFA loadings indicated that every measurement and scale objects are important and exceeded the smallest value standard of 0.70. Secondly, every hypothesis composite reliability surpassed 0.80. Thirdly, Hair et al. (2006) propose that an AVE ought to be 0.50 and beyond. As displayed in Table 1 and Figure 2, the AVE standards for all constructs are beyond 0.50.

Table 1 Validated convergent reliability, the authors expended Confirmatory Factor Analysis to survey the average variance extracted (AVE) and the combined reliability of the specific constructs.

**Table 1:** Result of average variance extracted (AVE) and reliability.

Measurement	Loading	Indicator Reliability	Error Variance <0.5	Sum of Variance	Compose Reliability	Ave. Variance Estimated
	>0.7				>0.8	>0.5
	**Tangibility**					
TAN1	0.845	0.7140	0.2860	1.1364	0.9096	0.9096
TAN2	0.823	0.6773	0.3227			
TAN3	0.898	0.8064	0.1936			
TAN4	0.816	0.6659	0.3341			

	**Reliability**					
REL1	0.857	0.7344	0.2656	1.9411	0.8868	0.6118
REL2	0.831	0.6906	0.3094			
REL3	0.777	0.6037	0.3963			
REL4	0.701	0.4914	0.5086			
REL5	0.734	0.5388	0.4612			

	**Responsiveness**					
RES1	0.853	0.7276	0.2724	1.5580	0.8608	0.6105
RES2	0.646	0.4173	0.5827			
RES3	0.870	0.7569	0.2431			
RES4	0.735	0.5402	0.4598			

	**Assurance**					
ASS1	0.910	0.8281	0.1719	0.7956	0.9415	0.8011
ASS2	0.916	0.8391	0.1609			
ASS3	0.841	0.7073	0.2927			
ASS4	0.911	0.8299	0.1701			

	**Empathy**					
EMP1	0.911	0.8299	0.1701	0.3163	0.9141	0.8418
EMP2	0.924	0.8538	0.1462			

CFA analysis of the study model was carried out and the outcomes specify that the model is suitable for the strength of the magnitude (Figure 2). Accordingly, Hair et al. (2006) suggested the need to reconsider the strength of concepts via convergent and discriminant validity tests. Thus, the criterion projected by Fornell and Larcker (1981) to compute discriminant validity was employed for the study. Discriminant validity can only be accomplished, when the square root of AVE for every hypothesis exceeds the relationship of that hypothesis and every additional concept (Fornell and Larcker, 1981). The maximum connection amid a specific construct and every additional concept is 0.8992; therefore, this rate is lesser when matched to the smallest square root of average variance extracted estimate (AVE) of every construct, which stands at 0.7814 (Table 2).

**Table 2:** Discriminant validity.

	TAN	REL	RES	ASS	EMP	S_SF	S_Loy
TAN	**0.8461**	0.6582∗∗	0.7663	0.7394	0.5668	0.6842	0.7805
REL		**0.7822**	0.6651	0.7021	0.6013	0.6574	0.6653
RES			**0.7814**	0.7041	0.4806	0.7683	0.6087
ASS				**0.8950**	0.6668	0.7331	0.7043
EMP					**0.8975**	0.5676	0.4801
S_SF						**0.8992**	0.7593
S_Loy							**0.8514**

∗p < 0.05; ∗∗p < 0.01; ∗∗∗p < 0.001.

The diagonal values represent the square root of the average variance extracted (AVE) of the specific construct.

Construct legend: S_Loy Student Loyalty; S_SF Student Satisfaction; REL Reliability; TAN Tangibility; RES Responsiveness; EMP Empathy; ASS Assurance.

Next, the discriminant validity was calculated using Pearson Correlation Matrix. As a standard, the discriminant validity dimension cannot be less than 0.90 (Campbell and Fiske, 1959). The coefficient of correlation can be measured via the correlation amid constructs. Results are presented in Table 2 indicating that the correlation coefficient is vastly connected and substantial.

Going by the outcomes of the analysis, it was established that the data are suitable in terms of construct reliability, discriminant validity and convergent validity. In a bid to respond to the enquiries in this study; to carry out hypotheses testing and accomplished the study's aims, a projected structure model was verified employing the structural equation modelling (SEM). In SEM, it illustrates the connection between perceived and unnoticed variables. The model suitability was appraised by scrutinising numerous suitable indices, which are chi-square/degree of freedom (χ^2^/df), Tucker Lewis Index (TLI), Goodness-of-Fit Index (GFI), Standardized Root Mean Residual (SRMR) Comparative Fit Index (CFI), and Root Mean Square Error of Approximation (RMSEA) (Table 3).

**Table 3:** The model fit.

Goodness of fit	SEMs Value	Recommendation Values	Remarks
Chi-square/Degree of Freedom (CMIN/DF)	2.568	≤3.00	Acceptable fit
Normed Fit Index (NFI)	0.962	≥.90	Good fit
Comparative Fit Index (CFI)	0.959	≥.90	Good fit
Incremental Fit Index (IFI)	0.971	≥.90	Good fit
Root Mean Squared Error of Approximation (RMSEA)	.074	≤.08	Good fit
Goodness of Fit (GFI)	.988	≥.90	Very Good fit

**Figure 3:**
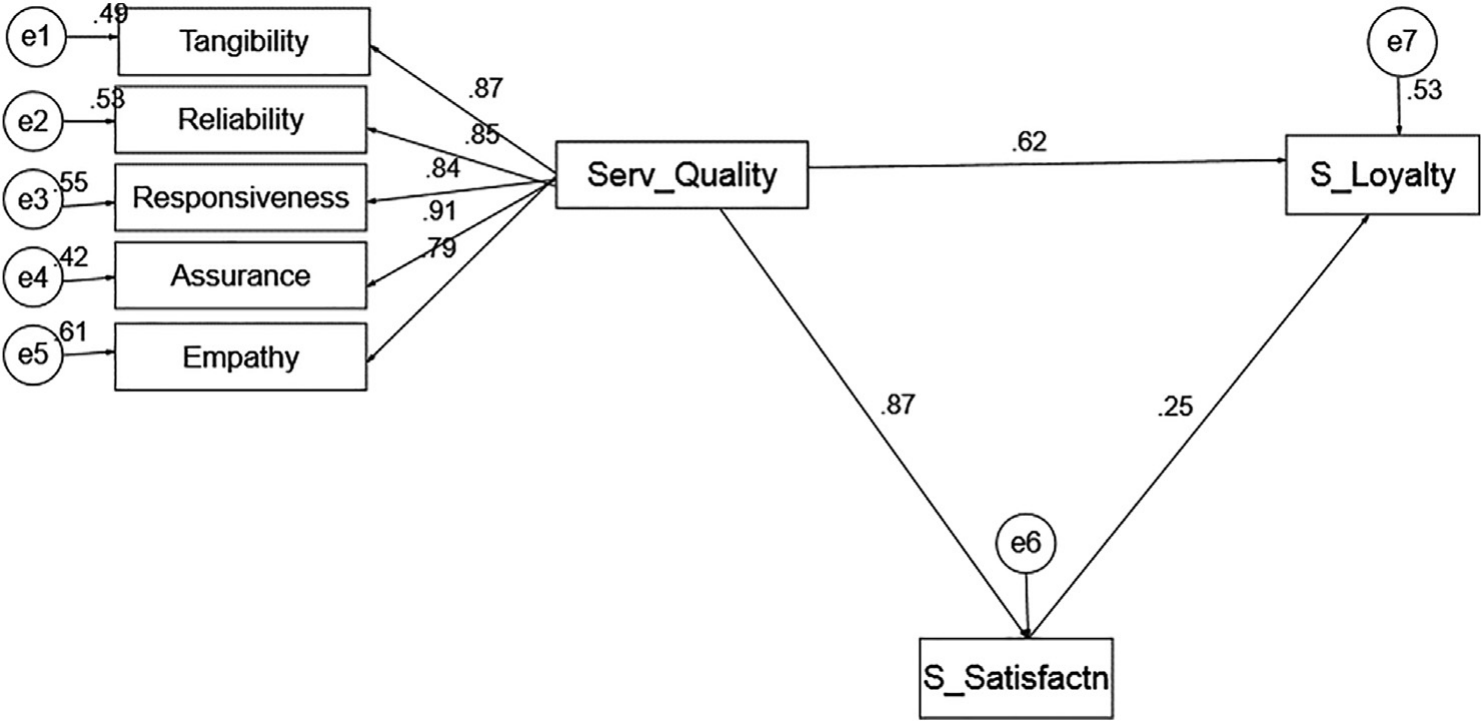
Strength of regression weights.

The outcomes in Table 3 prescribe that the rate of χ^2^ (71) = 187.179 and χ^2^/22 = 2.568 is between the satisfactory reach of 1 and 3 (Carmines and Mclver, 1981). The rate of Root Mean Square Error of Approximation (RMSEA) is 0.074, which is measured acceptable (less than 0.08) as recommended by Hu and Bentler (1999) and Brown and Cudeck (1993). Furthermore, the additional fit, CFI, NFI, GFI and TLI are beyond 0.90 (Bagozzi and Yi, 1998; Bentler and Bonnet, 1980). Moreover, the AIC is 221.649, which is adequately enough. Going by the results, it could be established that the entire fit indices are suitable. The results for standardised regression weights for all variables are specified in Figure 3 and Table 4. This shows that the strength of regression weights of pathways are reasonable.

**Table 4:** Standardized regression weights.

Independent		Dependent	Estimate	S.E.	C.R.	P	Label
S_Satisfactn	<---	Serv_Quality	.870	.033	28.615	∗∗∗	Sig
S_Loyalty	<---	Serv_Quality	.619	.065	9.287	.001	Sig
S_Loyalty	<---	S_Satisfactn	.249	.059	3.739	∗∗∗	Sig.
Tangibility	<---	Serv_Quality	.870	.033	28.634	∗∗∗	Sig.
Reliability	<---	Serv_Quality	.847	.036	25.854	∗∗∗	Sig.
Responsiveness	<---	Serv_Quality	.836	.033	24.748	.004	Sig.
Assurance	<---	Serv_Quality	.908	.037	35.142	.002	Sig.
Empathy	<---	Serv_Quality	.793	.047	21.157	∗∗∗	Sig.

∗∗∗Indicates that the relationship between the independent and the dependent variable is sig. at P < 0.01; S.E. = Standard Error; C.R. = Critical Ratio.

According to Arbuckle (2005), the square multiple relationships (R^2^) of a variable or measure is the percentage of its modification that is explained according to it predictors. Results are presented in Table 5.

**Table 5:** Squared multiple correlations.

Variables	Estimation
Empathy	.629
Assurance	.824
Responsiveness	.699
Reliability	.717
Tangibility	.756
S_Satisfactn	.756
S_Loyalty	714

The values of R^2^ indicate the change elucidated according to the variables as presented in the results Table 5:

It is estimated that tangibility explains 75.6 percent of the modification of students' loyalty
It is estimated that reliability explains 71.7 percent of the modification of students' loyalty
It is estimated that responsiveness explains 69.9 percent of the change of students' loyalty
It is estimated that assurance explains 82.4 percent of the alteration of students' loyalty
It is estimated that empathy explains 62.9 percent of the modification of students' loyalty.

The regression was carried out, which was verified in SEM concurrently. On the other hand, prior to conducting the analysis to conclude the hypotheses, the data were verified for normality, homoscedasticity, linearity and multi-collinearity.

**H**_**1**_**: attempts to examine the significant effect between the overall scopes of service quality and students' loyalty.**

In respect to the outcomes of the hypothesis testing, this paper discovered a positive and noteworthy effect between the quality of service and students' loyalty. The t-value of 9.287 (p-value = 0.000 < 0.05) indicated that service quality influences students' loyalty. Concerning the outcomes in Table 4, the standardised regression weight has calculated as β = 0.870, meaning that when service quality increases by 1 unit, students’ loyalty increases to 87%. This suggests that the greater the service quality, the more likely the student will display loyalty to the institution. Thus, this finding is in support of scholars who discovered the optimistic effect of service quality on the satisfaction of students and on the performance of the institution (Colgate and Danaher, 2000; Santos et al., 2020).

**H**_**2**_**: Attempt to examine the mediating influence of students' satisfaction amid service quality and students' loyalty.**

Having empirically verified the data, the result indicates that t-statistics is 3.739 and p-value is 0.014 which is (less than 0.050). Meanwhile, the β value is 0.2497, this indicates that, when changes in students' satisfaction increases by 1, the connection between service quality and students’ loyalty increases by 24.9%. This shows that when students are content with the services delivered, student loyalty is achieved. This is significant as continued satisfaction will lead to loyalty of student. This shows that student satisfaction mediate between service quality and student loyalty. The finding in this study is persistent with studies in marketing, which have revealed that satisfaction is the foremost precursor of customer loyalty (Narver et al., 2004; Ndubisi et al., 2009; Ibem and Aduwo, 2013).

## 4 Managerial implications

This research has emphasised numerous issues that are significant in sustaining higher educational institutions managers and practitioners in the education sector. These issues emphasise different areas that have been revealed to a have substantial impact on loyalty. Higher educational institutions’ managers should aim to provide quality service and certify that the students are satisfied to ensure student loyalty given the significant positive effect of service quality, learner or student satisfaction on loyalty. That is, if the student perceived the quality of service to be high, then the satisfaction level will be high and this will invariably lead to student loyalty. Evidently, service quality is central to learner satisfaction and student satisfaction precedes loyalty. This research has revealed that student loyalty is achieved only when services delivered by the educational institution satisfy the students. Therefore, the delivery of quality services should be targeted towards satisfying the student as this will help build the loyalty of the student to the institution.

## 5 Conclusions

Student's satisfaction is thought of as a significant variable that precedes student loyalty in the higher education sector. This research aimed to evaluate this concept by empirically examining whether service quality affect student loyalty and whether student satisfaction actually mediates the relationship between them. The hypotheses were analysed using SEM which showed that service quality dimensions significantly influence student loyalty. However, this relationship is mediated by student satisfaction. These results propose the need to integrate quality service in higher educational institution, as this will influence student satisfaction, which will invariably affect student loyalty. Since student loyalty tends to help have a positive behavioural intention towards the institution and a deep dedication to the HEI, which is likely to inspire the student to be engaged in the development of the institution. They can advance and encourage other individual to apply to the institution and also continue their postgraduate studies in the same institution if it is required. Therefore, HEI should accurately deliver services in a suitable and reliable way. They should be proactive in aiding the student by providing quick services, the environment of the institution should be conducive to learning at all times, the academic and the administrative staff should be well mannered and the HEI employees should give personal attention to the students and display special interest in them. All these practical measures will add to the improvement of student satisfaction and loyalty. Also, some of the possible consequences of student loyalty to the institution are enhancements in the competences of the university staff, adequate proficient internal marketing scheme, and improved total performance of the institution.

### 5.1 Limitations of the study

The limitation of this study is based on the fact that the study was carried out in one private university thus future research will need to apply it to other national and international Higher Education Institutions to acquire a greater understanding of service quality as a predictor of students satisfaction and student happiness.

## Declarations

### Author contribution statement

Borishade Taiye: Conceived and designed the experiments; Wrote the paper.

Ogunnaike Olaleke: Performed the experiments.

Odunayo Salau: Analyzed and interpreted the data.

Bolanle Motilewa, Joy I. Dirisu: Contributed reagents, materials, analysis tools or data.

### Funding statement

This work was supported by Covenant University Centre for Research, Innovation and Discovery.

### Data availability statement

Data will be made available on request.

### Declaration of interests statement

The authors declare no conflict of interest.

### Additional information

No additional information is available for this paper.

## Appendix A Supplementary data

RESEARCH QUESTIONNAIRE for this paper.docx RESEARCH QUESTIONNAIRE for this paper.docx
