# Supplementary material for: Assessing the relationship among service quality, student satisfaction and loyalty: the NIGERIAN higher education experience
Source: Heliyon. 2021 Jul 15;7(7):e07590. doi: 10.1016/j.heliyon.2021.e07590 (PMC8322287; doi:10.1016/j.heliyon.2021.e07590)
Supplement: RESEARCH QUESTIONNAIRE for this paper.docx [file mmc1.docx]

**RESEARCH QUESTIONNAIRE**

**Title:** Assessing the Relationship among Service Quality, Student Satisfaction and Loyalty: The Nigerian Higher Education Experience

**Authors**: Borishade Taiye T. *et al* (2021)

**Questionnaire**

**A = Biographical information of the respondents**

**B = Tangibility**

B1 = The institution lighting system in buildings is very attractive

B2 = The physical appearance of the institution’s buildings makes me consider the institution the first choice among others

B3 = The cleanliness of the campus satisfies me

B4 = I am pleased with the institution’s decorations of the atmosphere

**C = Reliability**

C1 = The institution has accuracy of records

C2 = The lecturers punctuality to class is very high

C3 = The institution service provider’s service is very reliable

C4 = The institution constantly provides quality teaching

C5 = The institution provides access to the internet always

**D = Responsiveness**

D1 = The lecturers are always available for assistance

D2 = Emergency management of lecturers is good

D3 = Emergency management of staff is very high

D4 = The service delivery is very efficient

**E = Assurance**

E1 = Research competence of lecturers is very high

E2 = Service competence of staff is very high

E3 = The institution has soundness of rules and regulations

E4 = The institution has strong security on campus

**F = Empathy**

F1 = Availability of learning resources is consistent

F2 = The level of individualized consideration is very good

**G = Student Loyalty**

G1 = I consider the institution first among other institutions

G2 = The institution is the first choice that comes to mind when making decision to further my studies

G3 = I am attached to the service of the institution

**H = Student Satisfaction**

H1 = I am absolutely happy with the institution

H2 = I am pleased with what the institution does for me

H3 = My experience with the institution is good

H4 = Overall, I am satisfied with the institution
